# Outcome of reverse shoulder arthroplasty secondary to rotator cuff arthropathy in a low-income population

**DOI:** 10.1186/s12891-023-07124-z

**Published:** 2024-01-02

**Authors:** Christine M. M. Silva, Gisele Façanha Diógenes Teixeira, Gabriella Cristina Coelho de Brito, Marco A. A. Lacerda, Francisco A. C. Rocha

**Affiliations:** 1 Orthopaedic Service, Shoulder and Elbow Group, Hospital Geral de Fortaleza, Fortaleza, CE Brazil; 2 Department of Internal Medicine, Faculdade de Medicina da Universidade Federal do Ceará, Fortaleza, Brazil

**Keywords:** Shoulder, Reverse shoulder arthroplasty, Arthroplasty, Inequality, Rotator cuff tear, Metabolic disease, Obesity, Smoking, Outcome

## Abstract

**Background:**

Reverse shoulder arthroplasty (RSA) is a valuable treatment for rotator cuff arthropathy (RCA) in developed regions. Socioeconomic issues impact access to specialized care and there is a lack of data on RSA outcomes in developing regions. We present our 24-month follow-up on RSA surgeries to treat RCA in our low-income population.

**Methods:**

Prospective evaluation of 26 patients subjected to RSA at Hospital Geral de Fortaleza-CE, Brazil, between January 2018 and December 2020. Literacy [>/≤ 8 school years(SY)] and income were documented. Outcomes considered pain (visual analogue scale; VAS) as well as SSV, SPADI, ASES, and UCLA scoring, and range of motion [forward flexion (FF); external rotation (ER)].

**Results:**

Patients were 68.5 ± 7.6 years-old with 16(61.5%) females; 65% had hypertension and 7 (26.9%) had diabetes. Over 90% declared < 900.00 US$ monthly family earnings and 10 (38.4%) patients declared ≤8 SY with > 80% exerting blue-collar jobs. Pain showed a significant reduction from baseline (8 ± 2) to 24 months (2.1 ± 2.3; *p* < 0.001). UCLA (10.3 ± 5.6 and 28.6 ± 7.2), ASES (16.7 ± 10.8 and 63.1 ± 28.4), SSV (326 ± 311 and 760 ± 234), and SPADI (98.3 ± 26.5) scores significantly improved from baseline to 24 months, achieving minimal clinically important difference. FF (89.2° ± 51.2° to 140.6 ± 38.3°) and ER (19.2° ± 22.5 to 33.4° ± 20.6°) significantly improved from baseline to 24 months (*p* = 0.004 and 0.027, respectively). There were 5 non-serious adverse events with one surgical revision. All patients returned to daily life activities.

**Conclusion:**

This is the first outcome report 2 years following RSA in a low-income population. Data indicate this procedure is justifiable regardless of socioeconomic issues.

## Background

Reverse shoulder arthroplasty (RSA) is currently the most commonly employed surgical treatment for rotator cuff arthropathy (RCA), especially among elderly patients. This procedure can also be indicated in patients with irreparable rotator cuff injuries, severe proximal humerus fractures, inflammatory arthropathies as well as for revision of primary arthroplasty being contraindicated when there is severe impairment of the deltoid muscle [1].

The diagnosis of RCA is primarily based on the presence of an irreparable rotator cuff tear (RCT) associated with degenerative changes in the glenohumeral joint and superior migration of the humeral head [2, 3]. The major objectives following RSA for RCA are pain relief and improvement in shoulder function, aiming to facilitate the return of patients to their daily life activities, thus limiting dependence on caregivers [4]. A remarkable increase in the number of RSA procedures has been seen in recent years, which is projected to continue in the near future [5]. These rising numbers underscore disparities, posing challenge**s** in developing regions. Limited access to specialized care has been associated with delayed diagnosis and worse surgical outcome. We have also previously commented that shortage of facilities for non-pharmacological treatments, including physiotherapy, negatively impact the quality of care provided to patients with rheumatic diseases in Brazil [6]. Furthermore, a recent study conducted in Germany and Switzerland emphasized that shoulder arthroplasty may facilitate rapid return to daily life activities including hygiene needs in elderly patients [4]. Therefore, delay in diagnosis, limitation of surgical access and absence of adequate physiotherapy could impact surgical outcomes in low-income patients. Data from large retrospective cohorts in the United States examined the influence of socioeconomic issues on surgical outcome following shoulder arthroplasty. For instance, it was shown that although all patients significantly improved following shoulder arthroplasty those with Medicaid support had lower pre and postoperative scores compared to individuals with Medicare or private insurance [7]. While a study conducted in 2022 did not find differences in outcome between patients living in the least or most disadvantaged neighborhoods [8], another analysis concluded that social disparities were linked to higher revision rates and adverse events following shoulder arthroplasty [9]. Similarly, using a distress community index, it was reported that individuals from distressed communities, regardless of race, had a higher risk of readmission and increased health care utilization following shoulder arthroplasty [10]. Intriguingly, a recent study found that individuals identified as White had higher rates of utilization of RSA for treatment of rotator cuff tear arthopathy compared to those of other races, excluding Black individuals [11]. On the other hand, a recent report showed excellent 4-year survivorship of both anatomic and reverse shoulder arthroplasty regardless of race, rural or urban residence, and income, used as proxies for socioeconomic disparities [12]. However, we are not aware of outcome data following RSA in developing regions. Our aim was to prospectively evaluate the results of two-year follow-up of RSA in patients suffering from RCA in our low-income, low-literacy population. For this purpose, patients were evaluated using the University of California at Los Angeles (UCLA) scale, the American Shoulder and Elbow Surgeons (ASES) score, the Shoulder and Pain Disability Index (SPADI), and the Simple Shoulder Value (SSV). The UCLA scale ranges from 0 to 35 and combines data from physical examination and patient reported outcomes with higher values meaning better function. The ASES score varies from 0 to 100 and combines physician and patient-rated information aiming to evaluate pain and function whereas the SPADI is a self-administered tool ranging from 0 to 130, a higher value meaning worst result, aiming to evaluate pain and disability. The SSV uses a patient’s subjective value as a percentage of a normal shoulder, which would be 100% [13–16]. These results are reported herein.

## Materials and methods

Between January 2018 and December 2020, thirty-two patients underwent RSA secondary to RCA at the Shoulder and Elbow Division of the Orthopaedic Service of the Hospital Geral de Fortaleza, CE, Brazil. Twenty-six out of those 32 patients were directly interviewed and subjected to clinical examination at baseline, 12 months, and 24 months follow up. Hospital records were reviewed for the collection of clinical data. The clinical protocol was approved by the local Ethics Committee (protocol number: CAEE 54107321.0.0000.5040) and all patients signed an informed consent prior to any procedure. Inclusion criteria encompassed patients with an irreparable rotator cuff tear associated with glenohumeral osteoarthritis subjected to RSA with over 2-years follow-up. This included individuals previously subjected to ipsilateral arthroscopic repair or muscle transfer as a treatment for rotator cuff tear. Exclusion criteria comprised patients with glenouhumeral osteoarthritis secondary to causes other than RCA, those requiring revision surgery as well as individuals with ipsilateral proximal humeral fracture, stress fractures or septic arthritis. Surgeries were performed by the two senior board-certified surgeons from our reference center, both having over 15-year practice in shoulder and elbow surgery (CMMS, MAAL). Briefly, patients were operated in the beach-chair position and subjected to a standardized deltopectoral approach followed by detachment of the subscapularis. Implantation of the prosthesis followed instructions of each manufacturer, namely: Lima SMR Reverse Modular Shoulder System (Lima Corporate S.p.A., Udine, Italy) or Equinoxe Reverse System (Exatech™, Warsaw, IL, USA). Whenever possible, the subscapularis tendon was reinserted. After surgery, the operated arm was kept in a Velpeau sling for 1 month. Although patients were encouraged to initiate physiotherapy 4 weeks after the surgery, most did not have access to appropriate facilities. Therefore, we did not evaluate adhesion to rehabilitation. Working activities were arbitrarily classified concerning predominantly as white or blue-collar jobs. Socioeconomic data included monthly family income using March 2020 as reference for conversion of Brazilian Real (R$) to US$ currency. Families earning < 3 minimum wages (roughly < 900.00 US$) monthly were considered of low-income [17]. Literacy was recorded as patients-declared years of school education [>/≤ 8 school-years (SY)] [18]. Pain intensity was calculated using a visual analogue scale (VAS). Outcomes did also include the UCLA, ASES, SSV, and SPADI instruments. The ASES, SPADI, and UCLA scales have been validated to Portuguese language [13–15]. Shoulder range of motion considered degrees of maximal active forward flexion (FF), and external rotation (ER). We also analyzed the mean values of our group with results considered of minimal clinically important differences (MCID) [19].

### Statistical analysis

Data are presented as means (standard deviation, SD) with definition of 95% confidence intervals (CI); comparison between groups was done using Friedman’s test. No imputation was done for missing data. The level of significance was set at 0.05 [20]. Analysis of data were done using IBM-SPSS v24, SPSS Inc.

## Results

### Patient demographics and socioeconomic data

There were 32 patients subjected to RSA during the study period. Reasons for not including six patients were death (1), lost to follow-up (3), and 2 did not attend the 24-month evaluation. Table 1 displays clinical and socioeconomic data for the 26 RSA procedures that completed the 24-month evaluation. Mean age at the time of evaluation was 68.5 ± 7.6 years (range, 54–82 years) with a female predominance [16 (61.5%)]. There were 7 (26.9%) and 5 (19.2%) patients with concomitant knee or hand osteoarthritis (OA), respectively, while 2 (7.6%) had generalized OA. Arterial hypertension was the most common comorbidity, present in over 65% of the patients, followed by 7 (26.9%) patients with type 2 diabetes. Most patients were considered low-income, with over 90% declaring monthly family earnings below 900.00 US$. Among the 20 patients that disclosed their education, none had a university degree, and 10 (38.4%) had less than 8 SY of formal education. Over 80% declared occupations that were arbitrarily classified as blue-collar jobs. Previous shoulder surgery was performed in 5 patients, as follows: proximal humeral fracture of the contralateral arm (1), RCT repair (3), latissimus dorsi transfer (1).

**Table 1:** Clinical characteristics of 26 low-income patients subjected to reverse shoulder arthroplasty secondary to rotator cuff arthropathy

Characteristic	N (%)
Female	16 (61.5)
Age	68.5 ± 7.6
Literacy	
≤ 8 years	14 (53.8)
> 8 years	12 (46.2)
Monthly family income	
US$ < 300	8 (30.7)
300 < US$ < 900	16 (61.5)
US$ > 900	2 (7.7)
Occupation	
Blue Collar	23 (88.4)
White Collar	3 (11.5)
Comorbidities	
Cardiovascular disease	2 (7.7)
Diabetes mellitus	7 (26.9)
Hypertension	17 (65.4)
Hand OA	5 (19.2)
Knee OA	7 (26.9)
Hand and Knee OA	3 (11.5)

Data represent mean (SD) and N(%);OA, osteoarthritis

### Clinical evaluation at 24-month follow-up

**Fig. 1:**
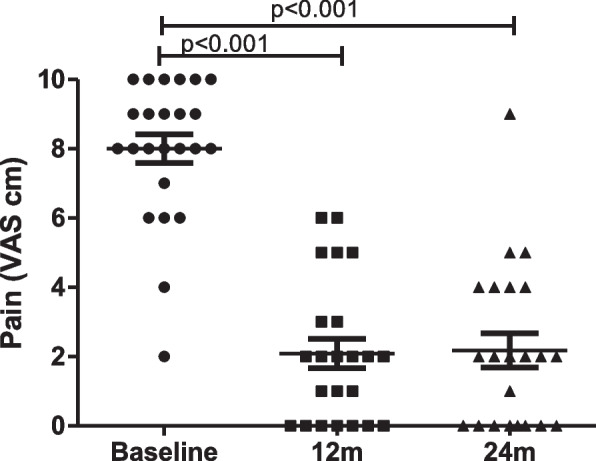
Variation of pain level (visual analogue scale, VAS) at baseline, 12 months (m) and 24 m following 26 reverse shoulder arthroplasty procedures in a low-income population. Data compared using Friedman’s test

**Fig. 2:**
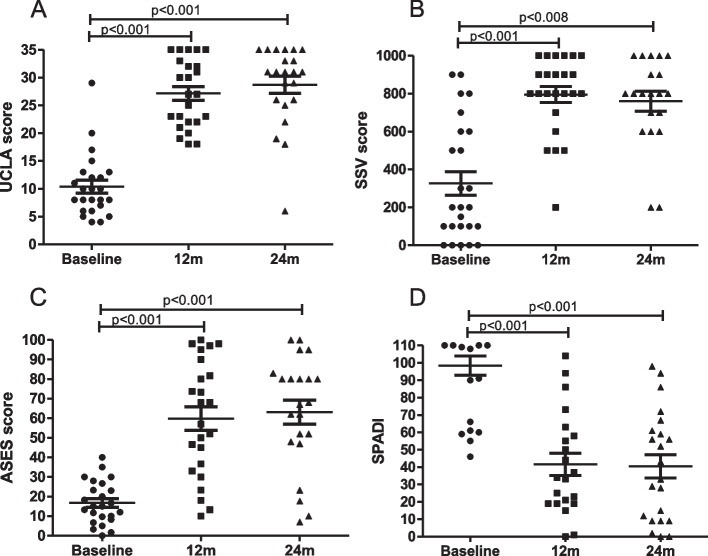
Variation of functional evaluation scales of 26 reverse shoulder arthroplasty procedures in a low-income population, including the University of California at Los Angeles (UCLA; **A**), Simple Shoulder Value (SSV; **B**), American Shoulder and Elbow Surgeons (ASES; **C**) and Shoulder and Pain Disability Index (SPADI; **D**) values at baseline, 12 months (m) and 24 m. Data compared using Friedman’s test

**Fig. 3:**
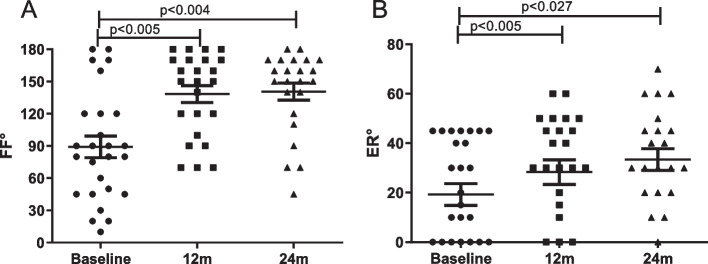
Variation of the shoulder range of motion regarding maximal degrees of forward flexion (FF) and external rotation (ER) at baseline, 12 months (m) and 24 m following 26 reverse shoulder arthroplasty procedures in a low-income population. Data compared using Friedman’s test

Mean pain reduction was significant following 1 year surgery (2 ± 2.0), being sustained at 24 months (2.1 ± 2.3), as compared to baseline (8 ± 2.0) (*p* < 0.001) (Fig. 1). Similar to pain reduction, evaluations made using functional scales were remarkably and significantly improved 1 year after surgery, being sustained after 2 years (Fig. 2a-d). The mean UCLA values at baseline and 24 months were 10.3 ± 5.6 and 28.6 ± 7.2, respectively, corresponding to 18.3 ± 1.6 mean ± SD points improvement. ASES values at baseline and 24 months were 16.7 ± 10.8 and 63.1 ± 28.4, indicating an increase of 46.4 ± 17.6 points. The SSV scores were 326 ± 311 and 760 ± 234 at baseline and 24 months, respectively, with an increase of 434 ± 77 points. SPADI scores decreased from 98.3 ± 26.5 at baseline to 40.4 ± 31.5 at 24 months, indicating a reduction of 57.9 ± 5 points. Values of functional improvement achieved MCID for pain reduction, ASES, SPADI and UCLA scores, according to previously proposed MCID results for these parameters of evaluation [12]. Range of motion was also improved, with FF increasing from 89.2° ± 51.2° at baseline to 140.6 ± 38.3° at 24 months (*p* = 0.004). The ER increased from 19.2° ± 22.5 at baseline to 33.4° ± 20.6° at 24 months, achieving statistical significance (*p* = 0.027) (Fig. 3a-b). Pain improvement was similar regardless of gender or being </ ≥ 65 years of age. However, pain relief in women had a trend for being lower, though not reaching statistical significance (data not shown). Survival rate of the prosthesis was 100% as no patient needed removal of the implant. There were adverse events in 5 RSA procedures (19.2%), as follows: superficial wound infection (1), neuropraxia of the ulnar nerve (2), luxation of the prosthesis (1) and 1 patient with periprosthetic fracture that also developed neuropraxia of the ulnar nerve. The superficial infection recovered with oral antibiotics. One of the patients with neuropraxia was subjected to transposition of the ulnar nerve leading to partial recovery. This patient had poliomyelitis during childhood, which may have influenced the occurrence of this adverse event. The other patient with neuropraxia received only clinical treatment with full recovery. One patient that sustained a dislocation was re-operated for open reduction with a constrict polyethylene revision. In the context of a total of 26 procedures, our revision rate amounted to 3.8%. This dislocation happened two months after the arthroplasty, being precipitated by his strenuous work on a farm, which involves lifting heavy objects. Unfortunately, he had access to our service only 3 months after the dislocation, owing to pandemic restrictions. We believe this delay influenced our failed attempt for closed reduction of the dislocation. The periprosthetic fracture occurred during the RSA procedure, involved the diaphysis of the humerus, and was approached with a revision humeral component. Patients were encouraged to initiate physiotherapy shortly after the surgery but most faced difficult access to appropriate facilities. Thus, we could not assess adhesion to rehabilitation, which was presumed to be very low.

## Discussion

This is the first study reporting surgical outcomes in a low-income population with a diagnosis of end-stage RCA that were subjected to RSA. Our data reveal a remarkable improvement in both pain and function scores at the 24-month follow-up, achieving values well over those considered of MCID [19]. The low-income characteristic of our present cohort was illustrated not only by the low values of declared mean monthly family income, but also by the relatively high percentage of individuals categorized as of low-literacy. Accordingly, a significant portion of our patients held blue-collar occupations, which are usually associated with lower salaries [6].

The relevance of this study lies largely in showcasing highly significant and clinically meaningful results following RSA in individuals facing socioeconomic challenges. Inequalities affect people in diverse ways, including lack of resources to afford care to perform basic daily life activities [6]. Few data report the incidence of RSA procedures in populations living in developing countries and, to the best of our knowledge, none focusing socioeconomic issues. A recent study reported 54,940 and 63,845 RSA procedures in 2016 and 2017, respectively, in the USA, while projecting an increase in these already remarkable numbers [5]. A report from an orthopaedic service in Brasília, Brazil, detailed the performance of RSA in 35 patients from 2011 to 2016. However, the data revealed information of 28 individuals, with only 11 patients where the indication for the RSA procedure was RCA [21]. Although living in the same country, inequalities exist in Brazil, which can be illustrated by a mean GDP/capita below US$ 4700.00 in our region as compared to over US$ 16,500.00 mean GDP/capita in Brasilia, Brazil [22]. Although hard to compare, previous data have shown that patients improve following RSA, regardless of socioeconomic issues [7–12]. Our results are thus encouraging vis-à-vis those reported with higher income cohorts. A study from Denmark reported data on a maximal 12-month follow-up of 566 patients subjected to RSA over a 6-year period. The study demonstrated clinically relevant improvement, regardless of patients being </≥ 65 years of age. Despite using other parameters of evaluations, the occurrence of adverse events was similar to our results. We believe patients with fewer socioeconomic disparities were included, but no socioeconomic data, besides being done in Denmark, a developed and affluent nation, were provided [23]. Outcome of arthroplasties is highly influenced by careful patient selection and expertise of the surgical team. Surgeries conducted by high-volume surgeons are associated with shorter hospital stay, lower cost and fewer adverse events, including a reduced revision rate [24]. Although still debatable, surgeons may be categorized based on their yearly arthroplasties numbers considering 0–4 as low-volume, 5–15 as medium volume, and > 15 as high volume [25]. Between January 2018 and December 2020, Dr. CMMS conducted 31 RSA, while Dr. MAAL performed 11 procedures, considering public and private practice. This would classify Dr. CMMS as a medium-volume surgeon and Dr. MAAL as a low-volume surgeon with relation to number of RSA procedures.

Besides causing pain, the functional limitation of the shoulder severely impairs balance and the ability to perform daily tasks, including personal hygiene. Notwithstanding, most are elderly patients, which makes the situation even more cumbersome [4]. Joint replacement is an expensive procedure, particularly in developing regions where other treatments may take precedence. Although life expectancy can be lower among low-income individuals in developing regions, the number of elderly people is expected to increase in the near future [6]. Being autonomous, particularly for patients with limited economic resources, is highly important, as they cannot afford caregivers, and access to public services for assistance with daily life activities is often challenging [4, 23]. We have previously shown, in a similar cohort, that surgical repair of patients with a RCT in our hands proved effective. However, our results in that study were not as robust when compared to data from wealthier cohorts. We believe that delayed access to specialized treatment have greatly influenced our results of RCT reparation, as compared to data from developed regions. Regarding the need for RSA, delayed RCT repair is linked to a higher chance of developing RCA. Thus, earlier referral and treatment for RCT might have impacted our results [1]. We may speculate that adequate timing of RCT repair in our hands could have reduced the need for replacement of the shoulder joint.

Our 24-month follow-up results for RSA were comparable to data from wealthier cohorts. Accordingly, Frankle et al. reported a mean 68.5 ASES score, while we had a mean 63.1 score at 24 months [26]. Similarly, Favard et al. reported a mean 130° of active anterior elevation in a French cohort with less than 5 years of follow-up following RSA for RCA, whereas our mean values for anterior elevation achieved 140.65° after 24 months [27]. We should also remark that some of our patients with mild or no compromise of elevation had significant loss of ER associated with radiographic end-stage glenohumeral osteoarthritis coupled with an irreparable rotator cuff tear, thus justifying indication of RSA. We also had an acceptable percentage of occurrence of adverse events, which is comparable to data from other cohorts, with only one patient needing revision of the implant. Although our evaluation was restricted to a two-year follow-up, revision surgeries, usually linked to infection and loosening issues, commonly occur shortly after surgery, usually within a 1–3 year period following the procedure [23, 28]. The fact that one of our patients had a dislocation of the prosthesis because of strenuous exercise linked to his job underscores daily challenges linked to socioeconomic issues. Restricting our indication for patients with RCA, which is probably the best candidate for this procedure, might have influenced our positive outcome. Indeed, revision rates were higher in younger patients subjected to RSA because of tumor, revision RSA, and fracture sequelae [28].

Our attempt to show clinical relevance was based on achieving results of MCID. It was previously proposed that MCID results for shoulder arthroplasty procedures, including RSA, implicate changes over 13.6 ± 2.3 and 8.7 ± 0.6 for the ASES and UCLA scores, respectively, as well as 20.6 ± 2.6 for the SPADI together with a minimum reduction of 1.6 ± 0.3 in pain using a VAS. Additionally, active FF and ER should improve by a minimum of 12° ± 4° and 3° ± 2°, respectively [19]. Mean improvement in our patients for the ASES and UCLA scores were 46.4 and 18.3, respectively, whereas SPADI and pain values had a reduction of 57.9 and 5.82 points, respectively. Values of FF and ER amplitude increased 51.4 and 14.1 at 24 months, as compared to baseline values. Although hard to compare with other series, our data are encouraging and substantiate that it is worth performing RSA in our patients, particularly given that our results meet previously proposed MCID values [19, 23, 26–28].

As outlined above, previous studies have shown that patients living in distressed communities, regardless of race, achieve comparable benefit to wealthier patients following RSA, although with higher revision rates, longer length of stay and more adverse events [10]. However, other studies reported similar outcomes regardless of social disparities [8, 12]. It was also recently reported that arthroplasties performed in low-income Hispanic individuals in the USA, when compared to non-Hispanics counterparts, had longer length of stay, costs and higher rates of acute kidney injury, blood loss, and need for blood transfusion. However, Hispanics were more frequently diabetic, with or without complications, which may have impacted the number of adverse events in this group [29]. Data from the UK revealed that patients from a socioeconomic deprived group subjected to RSA had more serious adverse events and a greater likelihood of surgical revision, as compared to less deprived individuals [30]. Although our low number of surgeries do not allow direct comparison with these findings, it is worth mentioning that our RSA outcomes align with those of wealthier cohorts, showing similar rates of adverse events and revision.

The present study was completely conducted within a public service that provides care for over 85% of our population, with no direct payments. One may wonder that those individuals with long delay prior to having access to a tertiary service hardly have rehabilitation facilities at their disposal [6]. The fact that we carefully selected our patients, restricting to those with RCA, which have a higher chance of good results, have probably contributed to our results, adding to justify the public investment. A major limitation of our study is the relatively low number of patients. We also cannot extrapolate our observations to other populations. However, publishing our two-year experience may help encourage surgeons working in services with similar inequalities to select patients that could benefit from RSA procedures.

## Conclusion

This is the first study reporting remarkable improvement of low-income patients living in a developing country subjected to RSA due to RCA. Results were similar to data reported from developed regions, which may justify this investment regardless of socioeconomic issues. Our data call attention for this alternative as a safe and effective therapy that significantly improve quality of life even in a low-income scenario.

## Data Availability

All data generated or analysed during this study are included in this published article.

## Abbreviations

ASES: American Shoulder and Elbow Surgeons score
BMI: Body mass index
CI: Confidence interval
ER: External rotation
FF: Forward flexion
IQR: Interquartile range
MCID: Minimal clinically important differences
RSA: Reverse shoulder arthroplasty
RCT: Rotator cuff tear
SY: School-years
SSV: Simple Shoulder Value
SPADI: Shoulder and Pain Disability Index
SD: Standard deviation
UCLA: University of California at Los Angeles score
VAS: Visual Analogue Scale.
